# Direct oral provocation test with beta-lactams in Brazilian children and adolescents

**DOI:** 10.1016/j.jped.2024.11.003

**Published:** 2024-12-03

**Authors:** Nathália Mota Gomes de Almeida, Mara Morelo Rocha Felix, Maria Inês Perelló Lopes Ferreira, Fábio Chigres Kuschnir

**Affiliations:** a Universidade do Estado do Rio de Janeiro (UERJ), Ciências Médicas, Rio de Janeiro, Brazil; b Universidade Federal do Estado do Rio de Janeiro (UNIRIO), Escola de Medicina e Cirurgia (EMC), Departamento de Clínica Médica, Rio de Janeiro, Brazil; c Hospital Federal do Estado do Rio de Janeiro, Serviço de Alergia e Imunologia Pediátrica, Rio de Janeiro, Brazil; d Universidade do Estado do Rio de Janeiro (UERJ), Saúde, Medicina Laboratorial e Tecnologia Forense, Rio de Janeiro, Brazil; e Hospital Universitário Pedro Ernesto, Serviço de Alergia e Imunologia, Rio de Janeiro, Brazil; f Policlínica Piquet Carneiro, Serviço de Alergia e Imunologia, Rio de Janeiro, Brazil; g Universidade do Estado do Rio de Janeiro (UERJ), Ambulatório de Reações Adversas a Medicamentos, Rio de Janeiro, Brazil; h Universidade do Estado do Rio de Janeiro (UERJ), Faculdade de Ciências Médicas, Departamento de Pediatria, Rio de Janeiro, Brazil

**Keywords:** Drug hypersensitivity, Beta-lactams, Children, Drug provocation test, Oral challenge, Beta-lactams hypersensitivity

## Abstract

**Objective:**

Beta-lactam (BL) allergy is considered a public health issue worldwide. To date, there is no consistent data on the direct Oral Provocation Test (OPT) for BL in Brazilian children and adolescents. This study's main objective is to describe the safety profile of direct OPT in this population.

**Method:**

A cross-sectional study was conducted with patients aged 1 to 17 years with a history of mild immediate or delayed reactions to penicillin. The European Network of Drug Allergy (ENDA) questionnaire was used. The authors performed OPTs with amoxicillin over five days. Continuous variables were described using their means and standard deviations. Bivariate analysis between test positivity and other study variables was performed using the Chi-square test, odds ratio, and their respective 95 % confidence intervals (CI 95 %). A *p*-value < 0.05 was considered significant.

**Results:**

In total, 54 OPTs were performed, four were positive (7.5 %) and one was considered inconclusive. All reactors were boys and had delayed reactions, with no severe reactions, and three showed symptoms on the first day of testing.

**Conclusion:**

>90 % of the sample was delabeled as allergic to BL. There were no severe reactions, confirming the safety of direct OPT in this age group. Among the reactors, 3 patients presented symptoms on the first day of testing before receiving the second dose and one had symptoms on 5 days, indicating that further studies are needed on the optimal duration of the OPT.

## Introduction

Antibiotics are responsible for up to one-third of reports of adverse drug reactions in hospital emergency departments, and beta-lactam (BL) is the group most frequently involved in these reactions in both the adult and pediatric populations.^1^, ^2^, ^3^, ^4^, ^5^

BL is the first-choice antibiotic for a significant portion of bacterial infections.^6^^,^^7^ Therefore, hypersensitivity reactions to these drugs are considered a public health problem, as they lead to the prescription of broad-spectrum antibiotics. This results in prolonged hospital stays, increased treatment costs, and increased bacterial resistance.^1^, ^2^, ^3^, ^4^^,^^6^^,^^8^, ^9^, ^10^, ^11^, ^12^

In the pediatric age group, the prevalence of BL allergy is estimated to be 10 % based on reports from caregivers. However, recent studies have shown a real prevalence lower than this value.^7^^,^^10^ It is estimated that up to 10 % of children experience some type of skin rash during antibiotic therapy. However, most of the time, the primary cause of these rashes in childhood is viral infections.^1^^,^^7^^,^^8^^,^^10^^,^^13^^,^^14^

Currently, the guidelines of the European Academy of Allergy and Clinical Immunology (EAACI) suggest that the diagnostic evaluation of suspected hypersensitivity reactions to BL should begin with anamnesis, followed by in vivo and in vitro tests, and subsequently, if necessary, the oral provocation test (OPT).^9^^,^^15^ The OPT is considered the gold standard due to its high sensitivity and specificity.^4^^,^^9^^,^^15^^,^^16^ This test consists of the supervised administration of the suspected drug in increasing doses until the therapeutic dose is reached.^9^^,^^16^ It should always be performed by an experienced doctor in an environment equipped to manage possible reactions to the medication.^16^^,^^17^

In the investigation of drug hypersensitivity reactions (DHR) to BL, it is suggested that skin tests include not only the suspected drug but also the main antigenic determinants of penicillins, which are the so-called major and minor determinants.^9^^,^^18^

Recent studies indicate that direct OPT, without skin tests, is safe and effective for delabeling patients previously classified as low-risk with a history of BL allergy, especially pediatric patients.^8^^,^^10^^,^^13^^,^^19^^,^^20^ There is no consensus yet on whether the OPT should be performed in a single day or on a prolonged basis.^7^^,^^21^, ^22^, ^23^

To date, there is no consistent data on the direct OPT for BL in Brazilian children and adolescents with a history of hypersensitivity reactions to these medications. The main objective of this study is to describe the safety profile of direct OPT and aspects related to its extended use in this population.

## Methods

### Population and sample

A cross-sectional study was carried out at the pediatric allergy and immunology outpatient clinic between June 2020 and March 2024. This is a specialized clinic located in a university center, equipped with the necessary medical materials for urgent care, as well as nursing staff available for the department. The study population consisted of children and adolescents aged 1 to 17 years, of both genders, with a history of mild cutaneous reaction to beta-lactams, who were seen at this clinic.

The sample of the study was composed of children and adolescents aged 1 to 17 years who were referred to the allergy center due to a clinical history of mild cutaneous reaction, seen according to the order of pre-scheduled appointments. The following were classified as mild reactions: generalized pruritus without associated symptoms; wheals without angioedema; diffuse maculopapular exanthema without systemic symptoms; contact dermatitis; localized wheals at the site of intravenous antibiotic infusion; localized reaction at the site of intramuscular antibiotic injection; and symmetric intertriginous flexural exanthema induced by drugs.

Patients with a history of severe reactions, such as wheals associated with angioedema, anaphylaxis, anaphylactic shock, and clinical manifestations suggestive of severe non-IgE-mediated cutaneous reaction (bullous lesions, mucosal involvement, and systemic symptoms), were excluded. Additionally, those with uncontrolled asthma, cardiovascular diseases, or an acute infection at the time of the test, as well as pregnant women, were also excluded.

### Data collection

The index reaction data were collected from the questionnaire developed by the European Network of Drug Allergy (ENDA), translated into Portuguese.^24^ In addition to the questionnaire, photographs of the index reactions taken by the caregivers at that time were used to help better define the cutaneous manifestation. Those who met the inclusion criteria underwent direct OPT with amoxicillin at a dose of 50 mg/kg/day, oral route, at least 4 week after the reaction. The OPT was open-label, meaning that family members and doctors were aware of the drug being administered, and it was conducted in a hospital setting under medical supervision. The patient received the antibiotic in two stages: initially, 10 % of the dose was administered, and after 20 min, if there was no reaction, the remaining 90 % was given. Then, the patient remained in the environment under medical supervision for 2 h. After this period, those who did not present a reaction were discharged to continue using the antibiotic at home for another five days. Patients also received second-generation antihistamines for use in case of a reaction and had direct phone access to the responsible physician. The OPT was immediately discontinued upon the onset of any objective reaction and appropriately treated.

At the end of these 5 days, a follow-up teleconsultation was carried out to collect general information and deliver the final report. In cases where reactions occurred, photos sent by the parents served as evidence, and a teleconsultation was scheduled so that appropriate guidance could be prescribed.

The result of the OPT was defined as positive or negative. Results were considered positive if the patient presented objective symptoms identified by the responsible physician and negative if no signs and/or symptoms were identified at the end of the test.

In addition to OPT, patients also underwent skin prick testing (SPT) to assess the presence of atopy. The following standardized allergenic extracts from the FDA Allergenic® laboratory were used: Dermatophagoides pteronyssinus, Dermatophagoides farinae and Blomia tropicalis. As a positive control, histamine (1 mg/mL) was used and as a negative control, the extract diluting solution. The main researcher, using the modified Pepys technique, performed the SPT. For each extract, individual disposable lancets (Alko do Brasil®) were used. The results were read 15 min after applying the extracts. A positive response was defined as a wheal size equal to or greater than 3 mm above the negative control.^25^

### Study variables

Patients were analyzed regarding sex, age, classification of the index reaction, as well as its duration and type of lesion. The presence of atopy, the time elapsed between the index reaction and the performance of the OPT, history of previous exposure to the antibiotic, and the type of compound involved were also evaluated.

Atopy was defined as the presence of sensitization to aeroallergens demonstrated by a positive skin test.^25^

Reactions occurring within 1 h after drug administration were classified as immediate reactions, and those whose symptoms manifested >1 h after the last administered dose were classified as delayed reactions.

The duration of the cutaneous manifestation after discontinuation of the antibiotic was divided into remission in <24 h, between 24 and 72 h, and >72 h. The time elapsed between the index reaction and the performance of the OPT was also evaluated, and it was divided into three categories: <1 year, 1 to 5 years, and >5 years. Cutaneous manifestations were classified as wheal when reported by caregivers as raised and erythematous plaques that completely subside within 24 h and as maculopapular exanthema (MPE) those represented by erythematous macules and infiltrated papules, not affecting mucous membranes.^6^

Participants were defined as having a history of previous exposure if they had used any beta-lactam before the index reaction.

### Statistical analysis

Continuous variables were described using their means and standard deviations (SD). Descriptive statistics were reported by their frequency. Bivariate analysis between test positivity and other study variables was conducted using the Chi-square test, odds ratio, and their respective 95 % confidence intervals (95 %CI). A p value < 0.05 was considered significant. All analyses were performed using the statistical software SPSS (SPSS Inc., Chicago, IL, version 23).

### Ethical aspects

The legal guardians of the patients signed the informed consent form prior to the beginning of the investigation. The study was approved by the Ethics and Research Committee, under protocol number 45232220.1.0000.5259.

## Results

During the study period, 81 patients were evaluated. Of these, 10 were excluded due to a history of severe reaction to BL, and 17 because their guardians did not consent to their children's participation in the study. In the end, 54 participants were included.

The median age was 5.8 years (min: 1; max: 16 years; SD: 3.472), 31 (57.4 %) were male, and the majority had no personal history of atopy (55.6 %) or family history of DHR (63 %). Regarding the index reaction, amoxicillin was the most involved antibiotic, present in 59.2 % of cases (Table 1).

**Table 1:** General characteristics of the sample and index reaction variables (*N* = 54).

Variables	N	(%)
Male sex	31	57.4
Presence of atopy		
Yes	24	44.4
No	30	55.6
Family history of atopy		
Yes	45	83.3
No	9	16.6
Family history of DHR		
Yes	20	37.0
No	34	63.0
Involved antibiotic		
Amoxicillin	32	59.2
Amoxicillin clavulanate	21	38.8
Benzathine penicillin	1	2.0
Previous exposure		
Yes	23	42.6
No	27	50.0
Does not recall	4	7.4
Classification of IR^a^		
Immediate	10	18.5
Delayed	44	81.5
Type of IR lesion		
Wheal	24	44.5
MPE^b^	29	53.7
Others	1	2.0
Duration of IR		
<24 h	18	33.3
24 to 72 h	19	35.2
>72 h	17	31.5
Time between IR and OPT		
<1 year	15	27.8
1 to 5 years	22	40.7
>5 years	17	31.5
Reexposure after OPT		
Not reexposed	25	51.0
Reexposed without reaction	22	44.8
Reexposed with reaction	2	4.0
Presence of cofactor		
Febrile infection	37	68.5
Afebrile infection	16	29.6
No cofactor	1	1.9
Main reasons for treatment		
URTI^c^	13	24.1
AOM^d^	12	22.2
Tonsillitis	11	20.4
Pneumonia	6	11.1
Others	12	22.2

a IR, index reaction.

b MPE, Maculopapular Exanthema.

c URTI, Upper Respiratory Tract Infection.

d AOM, Acute Otitis Media.

Delayed reactions and MPE were the most common reactions, corresponding to 81.5 % and 53.7 %, respectively. In approximately 70 % of cases, fever was present as a possible cofactor for the index reaction, and 50 % of the sample had no history of prior contact with BL (Table 1).

Regarding OPT, 59.2 % of patients were tested with the suspected drug. Only four (7.5 %) tests were positive, confirming the diagnosis of BL allergy.

Among the reactors, all were boys who presented a delayed reaction with mild manifestations and responded adequately to the use of second-generation antihistamines, with no occurrence of severe reactions.

**Figure 1:**
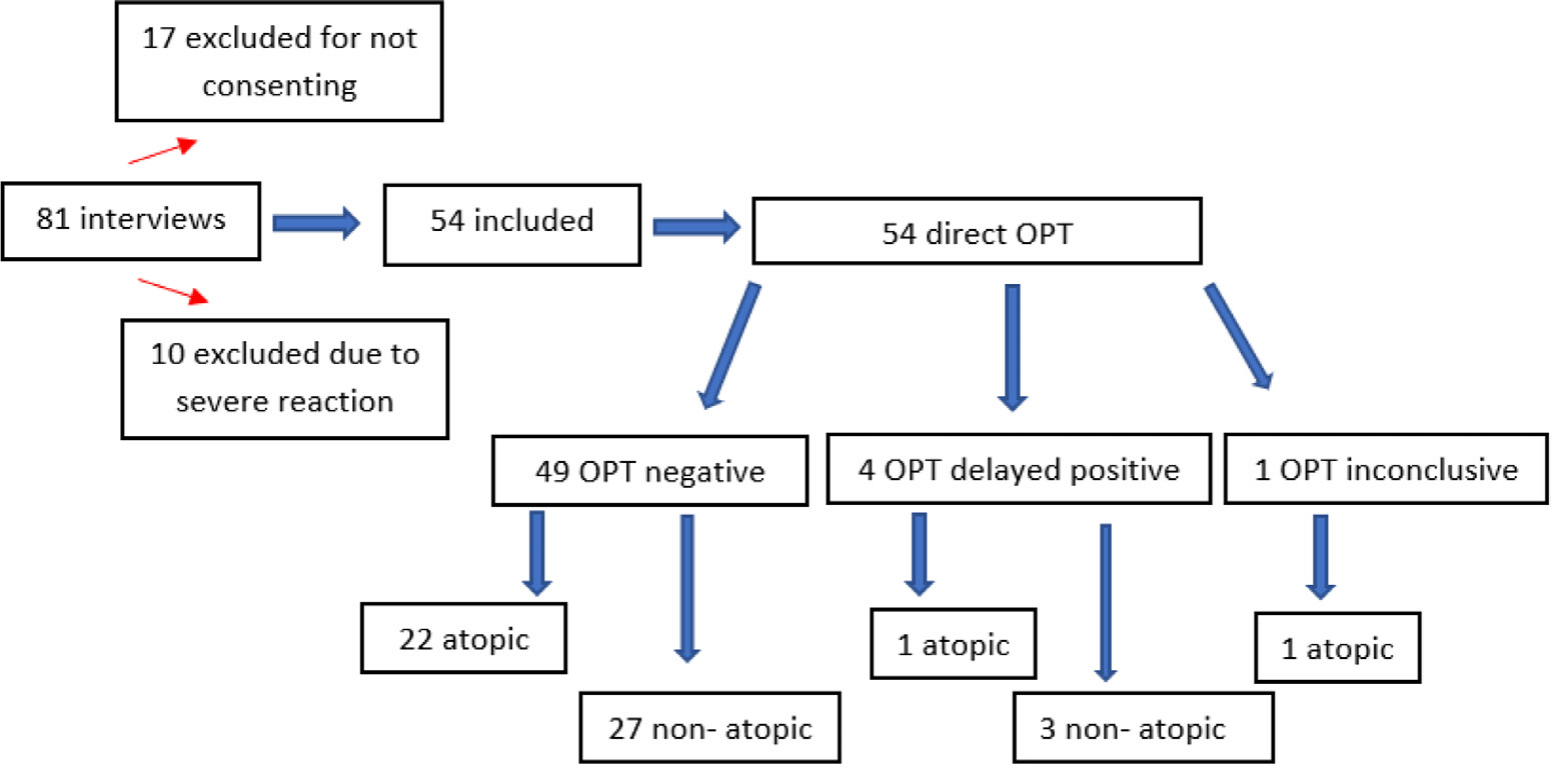
Flowchart of patients referred to the Allergy and Immunology Clinic between 06/20 and 03/24.

All patients who reacted OPT reproduced the cutaneous manifestation of the index reaction. Among the four reactors, three presented symptoms on the first day, before the administration of the second dose (2, 6 and 11 h after the first dose) and only one reacted on the fifth day. Thus, the authors observed that in 75 % of the cases, symptoms appeared <12 h after the first dose of the OPT. Only one (25 %) of the reactors had a positive SPT with aeroallergens (Figure 1).

There were no significant differences between test positivity and the main study variables (Table 2).

**Table 2:** Bivariate analysis between OPT positivity and study variables.

Variables	Positive OPT		Negative OPT		OR	95 % CI		*p*^c^
	N	%	N	%				
Sex								
Male	4	7.4 %	27	50.0 %	–	–	–	0.12
Female	0	0 %	23	42.6 %				
Atopy								
Yes	1	1.9 %	27	50.0 %	0.39	0.038	4.02	0.62
No	3	5.6 %	23	42.6 %				
Family history of atopy								
Yes	2	3.7 %	43	79.6 %	0.16	0.020	1.35	0.12
No	2	3.7 %	7	13.0 %				
Family history of DHR								
Yes	1	1.9 %	19	35.2 %	0.54	0.053	5.61	1.00
No	3	5.6 %	31	57.4 %				
Involved antibiotic								
Amoxicillin	3	5.6 %	30	55.6 %				
Amoxicillin + Clavulanate	1	1.9 %	20	37 %				
Previous exposure								
Yes	1	2 %	21	42 %	2.47	0.210	29.2	0.58
No	2	4 %	26	52 %				
Classification of IR^a^								
Immediate	0	0 %	10	18.5 %	0.80	0.69	0.91	0.42
Delayed	4	7.4 %	40	74 %				
IR Lesion								
MPE^b^	2	3.8 %	27	50.9 %	0.81	0.10	6.26	1.00
Wheal	2	3.8 %	22	41.5 %				
IR Cofactor								
Yes	3	5.6 %	34	64.2 %	1.32	0.12	13.7	1.00
No	1	1.9 %	15	28.3 %				

a IR, Index Reaction.

b MPE, Maculopapular Exanthema. OR, Odds Ratio; 95 % CI, 95 % Confidence Interval.

c Chi-square Test.

One patient presented MPE three days after the end of the OPT and was classified as inconclusive OPT. For this reason, it was suggested to the parents to repeat the procedure, but there was no consent and thus the recommendation to avoid BL was maintained.

## Discussion

A significant portion of the sample had a negative OPT for BL, consistent with results reported in other regions of the world, which also found percentages above 90 % for this outcome.^3^^,^^7^^,^^10^^,^^13^^,^^19^^,^^20^

Most of the study participants presented mild delayed reactions after using amoxicillin to treat upper respiratory infections, similar to those reported in other recent studies conducted in different countries.^3^^,^^7^^,^^13^^,^^10^, ^11^, ^12^^,^^19^^,^^20^

The authors demonstrated that relying solely on clinical history in children and adolescents is not sufficient to predict who will react to the OPT, highlighting that the diagnosis of allergy to these antibiotics is overestimated when using only this investigative tool. Obtaining detailed information about the reaction is not always possible due to the inaccuracy of the data reported by caregivers.

In this sample, 50 % of the investigated individuals denied previous use of the involved drug. Among the four patients who had their allergy confirmed, three had a history of previous exposure. In the case of the patient who had their allergy confirmed without prior exposure, several hypotheses regarding sensitization can be considered, such as intrauterine exposure or through breast milk, the possibility of cross-reactivity with other BL, or even an error in the report during anamnesis.

Of the total children indicated for the OPT, 21 % of parents did not consent to the investigation, either due to fear of possible reactions or because they did not consider such a diagnosis important. This data highlights the importance of educating the public and physicians about the safety of tests performed by experienced professionals in controlled environments, as well as the negative consequences of exposing children to broad-spectrum antibiotics, often unnecessarily due to an incorrect diagnosis. Educating the population about the risks and updating healthcare professionals on the topic play an important role in improving public health and significantly reducing the issue of growing bacterial resistance.^2^^,^^26^

Studies are scarce in Brazil on the profile of antibiotic prescriptions in our country, however, a recent publication evaluated this data between January 2014 and July 2021. During the study period, amoxicillin, azithromycin, and cephalexin accounted for 2/3 of all prescriptions, with a slight decrease in amoxicillin prescriptions after the COVID-19 pandemic.^27^

In the 1990s, the Brazilian Ministry of Health published a document on the investigation of DHR to BL due to the resurgence of acquired syphilis observed in recent decades. The document advocated for the safety of this investigation based on the low incidence of anaphylaxis caused by these medications.^28^ Jares et al.^5^ evaluated 11 Latin American countries regarding drug-induced anaphylaxis and demonstrated that non-steroidal anti-inflammatory drugs are the most involved, followed by BL. Collectively, these studies are crucial to understanding the risks of broad-spectrum antibiotic use, such as the low incidence of anaphylaxis caused by BL. They highlight the ongoing need to invest in research that guides public health policies and safe clinical practices in antibacterial prescription.

The present study investigated both mild immediate and delayed reactions to BL, with no severe reactions occurring during the OPT in either group, which supports recent studies on the safety of direct OPT.^3^, ^4^^,^^10^^,^^12^, ^13^, ^14^^,^^19^, ^20^^,^^23^ Recently, a meta-analysis included 28 studies, totaling 8334 patients up to 18 years old who underwent OPTs for BL, with a positivity rate of 5.2 % and a very low frequency of severe reactions, reinforcing the safety of direct OPT in this age group.^29^

All study participants completed the five-day OPT, and among the four positives, three reacted on the first day of the test. This finding suggests that a one-day OPT is capable of identifying most patients allergic to BL. However, there is currently no consensus on the ideal duration for the OPT.

Petersen et al. evaluated 305 patients aged 0 to 18 years with suspected BL allergy using a five-day OPT. The authors concluded that the prolonged test increases its sensitivity and the parents' confidence in future exposure.^23^ This opinion is also supported by Celik et al.^22^ Additionally, since most reactions are delayed, a prolonged OPT would mimic the actual treatment duration.^3^^,^^7^^,^^21^^,^^22^^,^^25^ On the other hand, Mill et al. identified an NPV of 89.1 % for a one-day OPT,^13^ and Caubet et al.^14^ suggest that two days are enough to adequately investigate these reactions. To date, no studies are compared extended OPT with short-term OPT.

In this study, 75 % of patients who had a positive OPT had a reaction on the first day of the test and 25 % on the last day. In this study, the washout period was not performed.^9^^,^^15^ This recommendation is based on evidence that the initial dose can cause reactions up to 48 h to 7 days later, therefore this single patient who reacted on the fifth day of the test could have reacted even if the OPT was of short duration.

There are still areas without consensus regarding DHR, such as the distinction between immediate and delayed reactions. The classification used in this study aligns with most studies cited in this text,^3^^,^^7^^,^^10^^,^^13^^,^^21^^,^^23^ although some authors define immediate reactions as those occurring within 6 h after the last exposure, while delayed reactions may manifest at any time after 1 h post-dose, resulting in the temporal overlap. As noted by Blanca-Lopez et al., this definition is controversial, and the ideal classification has yet to be established.^30^

In the present study, 72 % of patients undergoing the OPT had experienced the index reaction more than one year ago. Studies show that patients with IgE-mediated DHR may lose this sensitization over time, with 30 % of patients potentially losing it within one year.^4^^,^^9^, ^10^, ^11^ Therefore, efforts should be made by responsible organizations to ensure that this investigation is conducted as early as possible, thereby providing a realistic scenario regarding DHR to beta-lactams.

No significant statistical association was found between the OPT result and study variables such as sex, age, and atopy. These findings were similar to those of other studies.^7^^,^^12^^,^^23^

In this sample, 68.5 % had a fever as a possible cofactor during the index reaction. This suggests an important role of viruses as a cause of the rash.^3^^,^^7^^,^^8^^,^^14^^,^^22^ Both viral infections and delayed DHR involve the activation of T lymphocytes, potentially causing symptoms through the direct action of each of these agents or their immunological interaction.^3^^,^^8^^,^^13^ The presence of fever in the reactions generates another confounding factor in the clinical history, which is the concomitant use of antipyretic medications, which become suspected of causing DHR depending on the chronology of events.

After the testing period, all patients who presented negative OPT (49) were contacted via telephone to assess their history of re-exposure to BL. Of these, 22 (44.8 %) were re-exposed without any reaction, 25 did not receive a new antibiotic prescription, and two had cutaneous manifestations upon re-exposure post-OPT. Both had the index reaction during the use of amoxicillin with clavulanate and did not react to the OPT with amoxicillin. Because re-exposure occurred with the combination of amoxicillin and clavulanate, it is possible that the drug involved in these cases is clavulanic acid. Although it is considered rare, there are reports in the literature of DHR to clavulanic acid.^1^^,^^30^ Unfortunately, the patients with this suspicion in the sample were not subjected to a new test, making it impossible to rule out the possibility of a new cutaneous manifestation triggered by an infection

The present study has some limitations. Firstly, it was initiated in June 2020, during the COVID-19 pandemic, which hindered access to more patients due to some refusing to be exposed to hospital environments at that time. Additionally, the source of information was the history collected from caregivers, which may introduce memory biases. Despite its small sample size and being conducted at a single tertiary center, which may limit the generalization of the results, this is the largest national study on the safety and efficacy of direct OPT in children and adolescents. Furthermore, standardized instruments were used and international recommendations were followed for carrying out the OPT.

In conclusion, this study showed that >90 % of the tested children and adolescents were delabeled as allergic to BL, indicating an overdiagnosis of allergy to these drugs following other publications on the topic. There were no severe reactions, confirming the results of international studies on the safety of direct OPT.

## Funding

This research did not receive any specific grant from public or commercial funding agencies.

## Conflicts of interest

The authors declare no conflicts of interest.
